# A Case of Epidermal Growth Factor Receptor-Mutated Non-Small-Cell Lung Cancer: Multi-Line Treatment and Resistance Mechanisms

**DOI:** 10.7759/cureus.33577

**Published:** 2023-01-09

**Authors:** Ana Raquel Teixeira, Rute Fernandes, Ana Rodrigues

**Affiliations:** 1 Medical Oncology, Instituto Português de Oncologia do Porto, Porto, PRT

**Keywords:** resistance mechanisms, egfr tyrosine kinase inhibitor, egfr mutation, lung adenocarcinoma, non-small cell lung cancer

## Abstract

In the non-small-cell lung cancer (NSCLC) subtype, adenocarcinoma is the most common histology. The choice of first-line treatment depends on the mutational status.

We present a case of a 54-year-old non-smoker woman with lung adenocarcinoma and extensive metastatic disease at diagnosis. The genetic analysis demonstrated a sensitizing epidermal growth factor receptor (EGFR) exon 19 mutation and she began treatment with a first-generation EGFR tyrosine kinase inhibitor (TKI), erlotinib. Due to successively acquired resistances and disease progression, six treatment lines were pursued, including third-generation EGFR TKI and chemotherapy. The patient accomplished an overall survival of 47 months.

This case emphasized the importance of monitoring tumor mutational alterations, allowing us to implement a multi-line treatment. Targeted therapy in advanced NSCLC largely improved the overall survival of these patients.

## Introduction

The most common lung cancer subtype is non-small lung cell cancer (NSCLC), accounting for more than 80% of all cases [1]. Adenocarcinoma is the most frequent histologic type of NSCLC [2]. After the diagnosis of advanced lung adenocarcinoma, guidelines suggest testing for oncogenic drivers, namely epidermal growth factor receptor (EGFR) mutations, first described in 2004, rearrangements involving the anaplastic lymphoma kinase (ALK) and c-ROS oncogene 1 (ROS1), Kirsten rat sarcoma viral oncogene homolog (KRAS), and BRAF mutations [2,3]. Among activating EGFR mutations, 45% are in-frame deletions of exon 19 around the LREA motif and 40% are p.L858R point mutations in exon 21 [4]. EGFR tyrosine kinase inhibitors (TKI) have been established as a treatment option, demonstrating benefits versus platinum-based chemotherapy, in patients with sensitizing EGFR mutations [3].

Most patients treated with first or second-generation EGFR TKI develop resistance after 9-14 months of treatment [5]. Frequently, the involved resistance mechanism is the p.Thr790Met (T790M) mutation in exon 20 of EGFR [5]. Osimertinib is a third-generation EGFR TKI that overcomes T790M resistance mutation, with higher median overall survival versus other EGFR TKIs in previously untreated advanced NSCLC EGFR-mutant [6].

Despite third-generation EGFR TKI efficacy, treatment resistance is also a concern and includes diverse mechanisms (EGFR-dependent and EGFR-independent pathways) [5]. The EGFR-dependent (“on-target” resistance) accounts for alterations related to EGFR signaling, such as T790M loss which occurs in 50-60% of patients treated with first or second-generation TKI [7]. In EGFR-independent pathways (“off-target” resistance) other parallel mechanisms are responsible, for instance, MET amplification or histological transformation to small-cell lung cancer (14% in first-line and 4-15% in second-line treatment with osimertinib) [7].

Patients with disease progression require changes in EGFR TKI therapy so, if possible, additional biopsies or circulating tumor-DNA (ctDNA) tests should be performed to identify the resistance mechanism [4].

In this case report, we present a case of metastatic EGFR-mutated NSCLC with a long survival after a multi-line treatment that included different EGFR TKI and chemotherapy regimens.

## Case presentation

We present the case of a 54-year-old female with right arm pain that radiated to the neck. She had no relevant comorbidities and no history of smoking. Physical examination revealed an enlarged right supraclavicular fossa and cervical lymph nodes; ultrasonography confirmed an approximately 10 mm mass.

**Figure 1:**
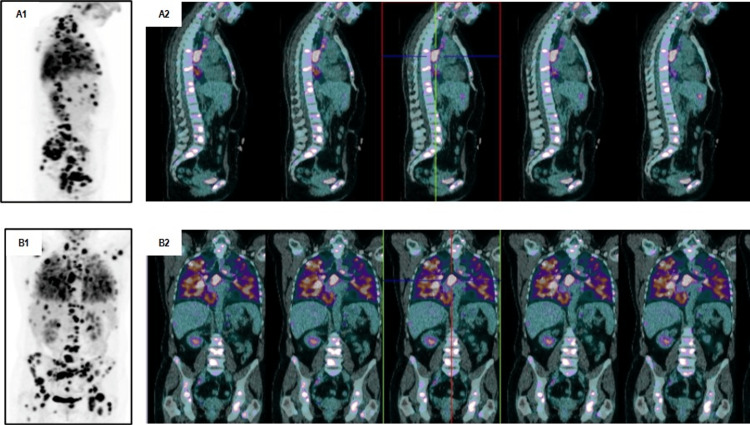
Positron-emission tomography-computed tomography (PET-CT) scan images demonstrating extensive metastatic disease at diagnosis with high maximum standardized uptake value (SUV) in lungs, lymph nodes (supraclavicular, paratracheal, prevascular, aortopulmonary, subcarinal and hilar bilateral), liver, left suprarenal gland and bone (all spine, sacred bone, pelvis, sternum, scapulas, right clavicle, ribs, humerus, and femurs). A1) maximum-intensity-projection images on sagittal plane; A2) PET/CT sagittal view; B1) maximum-intensity-projection images on coronal plane; B2) PET/CT coronal view.

Additionally, a positron-emission tomography-computed tomography (PET-CT) scan showed a right superior lobe lung mass (25 mm) and multiple smaller bilateral nodules. The biopsy of the largest lung mass presented adenocarcinoma (CK7 and TTF1 positivity, CK20 and thyroglobulin negativity), favoring lung origin, PD-L1 <1%. Tissue next-generating sequencing (NGS)-based genetic testing revealed an EGFR exon 19 mutation (c.2235_2249del). A PET scan confirmed advanced disease with several metastases: lung, lymph nodes, liver, left suprarenal gland, and bone (Figure 1). So, she was diagnosed with lung adenocarcinoma stage IV (cT4N3M1b).

**Figure 2:**
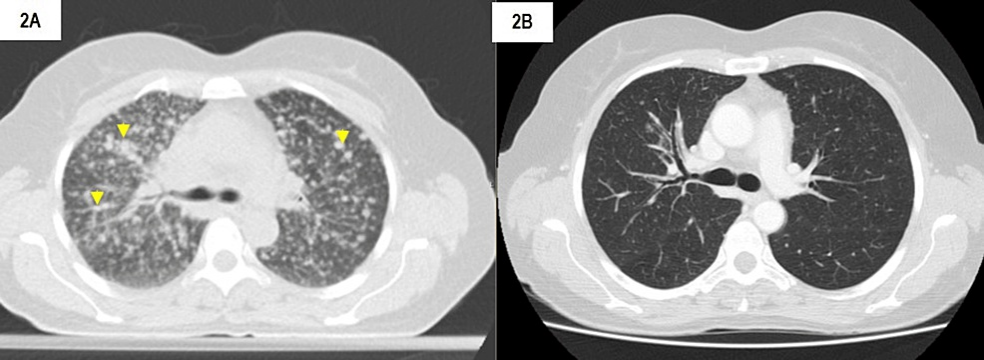
2A) Positron-emission tomography-computed tomography (PET-CT) scan image demonstrating lung metastasis at diagnosis (02/10/2017); 2B) thoracic CT scan after beginning erlotinib, showing disease response (30/11/2017); (yellow arrows: example of pulmonary lesions).

This patient had an ECOG score of zero and began treatment with erlotinib (150 mg q.d.), a first-generation EGFR TKI, in October 2017. Due to bone metastases, she also began treatment with zoledronic acid 4 mg monthly. After a month of treatment, the patient presented a disease response with the disappearance of most lesions, without any adverse effects (Figure 2).

After 14 months of erlotinib therapy, a thoracic CT scan showed pulmonary disease progression. CtDNA testing was performed and an exon 20 mutation (p.T790M mutation) was identified, so second-line treatment with the third-generation EGFR TKI osimertinib (80 mg q.d.) was proposed.

In December 2019, at 12 months of osimertinib therapy, lung disease had progressed; ctDNA analysis showed the disappearance of the p.T790M mutation. Third-line treatment with carboplatin AUC 6 and pemetrexed 500 mg/m2 every 21 days was begun.

**Figure 3:**
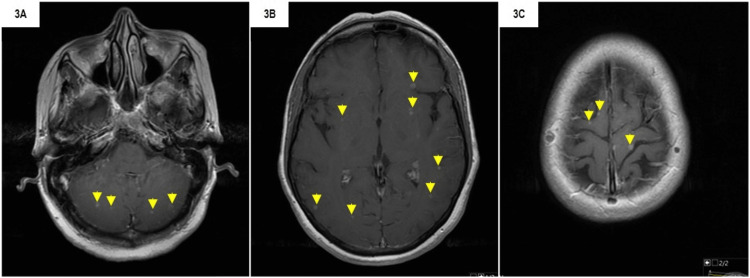
Cerebral MRI showing miliary cerebral metastasis. 3A-3C) multiple hyperintense lesions in T1-weighted with gadolinium and in different axial planes; yellow arrows: example of cerebral lesions

After three chemotherapy cycles, the patient presented with dizziness and unsteady gait, and cerebral imaging was performed, showing miliary cerebral metastases (Figure 3). Concomitantly, the patient presented with a progression of pulmonary disease. The multidisciplinary team considered the best treatment consisting of three-dimensional conformal holo-cranial radiotherapy (20 Gy/5 fr) and a fourth-line systemic treatment with docetaxel 100 mg/m^2^ every 21 days for six cycles.

In August 2020, a month after chemotherapy ended, a thoracic CT scan revealed an increasing size and number of pulmonary metastases. Exon 20 p.T790M mutation reappeared on ctDNA and we proposed an osimertinib repeat treatment.

**Figure 4:**
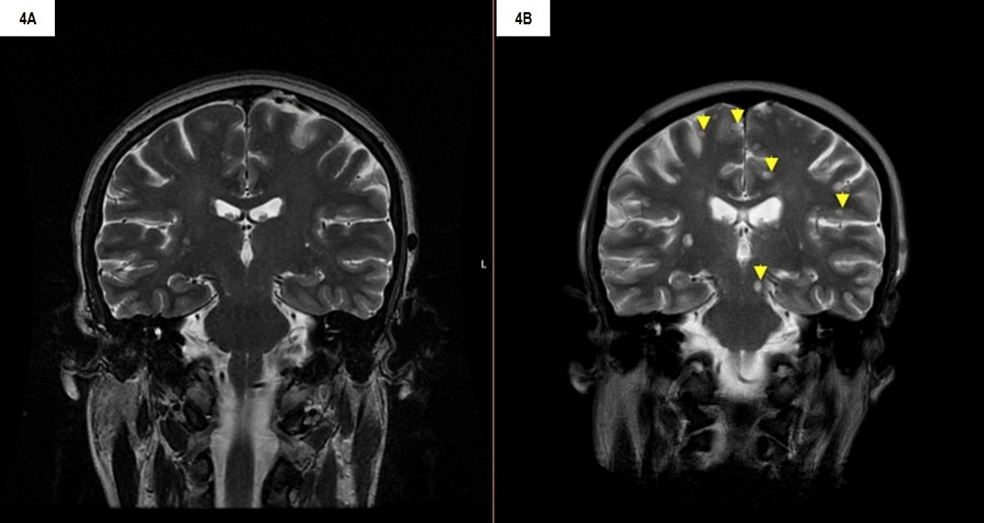
Cerebral magnetic resonance imaging showing cerebral disease progression—comparison between March 2020 (4A) and July 2021 (4B); yellow arrows: new cerebral lesions.

This drug achieved three months of disease control before further disease progression. To clarify the disease resistance mechanism, a repeat lung biopsy was performed, which confirmed the disappearance of exon 20p.T790M mutation on ctDNA. The lung biopsy was compatible with lung adenocarcinoma and genetic analysis revealed c.2235_2249del on exon 19, as at initial diagnosis. Treatment was changed to gemcitabine 1250 mg/m2 monotherapy every 28 days. The patient completed seven cycles of gemcitabine but experienced significant clinical deterioration, notably in the lungs, thoracic lymph nodes, and central nervous system (Figure 4). In July 2021, palliative care was offered until the patient died one month later.

## Discussion

**Figure 5:**
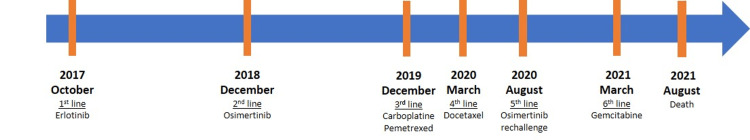
Timeline of the patient’s medical history.

In recent years, advances in the treatment of NSCLC have allowed patients to complete longer and more complex treatment strategies with multi-line treatments (Figure 5). Due to targeted therapy, it is now possible for patients with advanced or metastatic lung adenocarcinoma to achieve longer life, our case report afforded the patient 47 months of survival.

Third-generation EGFR TKIs, such as osimertinib, are presently the preferred first-line treatment of EGFR-mutant lung cancer, which differs from our report [4,8]. The mechanism of acquired resistance when osimertinib is used as first-line treatment is unknown in 40-50% of cases [7]. Liquid biopsy with a real-time polymerase chain reaction (PCR) test is commonly used to detect the most common genetic alterations, as previously described. However, as acquired resistance mechanisms to osimertinib are heterogenous, perhaps the application of NGS panels to analyze ctDNA is the best approach as it allows the detection of actionable genomic alterations [9]. For instance, in 10-30% of patients with EGFR-mutant NSCLC treated with first-line osimertinib, the acquired resistance mechanism is the development of MET amplification [10]. In the INSIGHT 2 study, this amplification could be detected by fluorescence in-situ hybridization (FISH) in tissue biopsy and/or NGS in liquid biopsy [10]; another important resistance mechanism is small-cell lung cancer transformation (3-10% of all EGFR TKI cases) [9]. Due to the frequent use of liquid biopsy and the absence of re-biopsy at the time of progression, this incidence may be underestimated [9].

After disease progression during treatment with osimertinib, chemotherapy remains the standard approach [2,4]. Regarding our case report, after third-line treatment with platinum (first chemotherapy line), adding nintedanib to docetaxel could be an option, as this can be an effective combination in patients with advanced NSCLC previously treated with one line of platinum-based therapy [11]. In the LUME-Lung 1 trial, nintedanib plus docetaxel was associated with longer progression-free survival and overall survival in adenocarcinoma patients, compared to docetaxel plus placebo [11].

Osimertinib rechallenge in monotherapy or combination may be considered [4,12]. There are ongoing clinical trials that aim to investigate how to prevent the emergence of resistance to third-generation EGFR TKI by combining TKI, chemotherapy, and/or immunotherapy as front-line treatment [7].

## Conclusions

The advanced NSCLC paradigm had changed due to targeted therapy and with benefits for the overall survival of these patients. Precision oncology brings new challenges to our clinical practice and this case demonstrates the importance of following the tumor mutational dynamics during a multi-line treatment, as the treatment strategy (TKI versus chemotherapy) may change depending on disease evolution.
